# GSK3β signaling is involved in ultraviolet B-induced activation of autophagy in epidermal cells

**DOI:** 10.3892/ijo.2012.1620

**Published:** 2012-09-05

**Authors:** YANG YANG, HAIPING WANG, SIYING WANG, MEI XU, MEI LIU, MINGJUN LIAO, JACQUELINE A. FRANK, SABAL ADHIKARI, KIMBERLY A. BOWER, XIANGLIN SHI, CUILING MA, JIA LUO

**Affiliations:** 1 Department of Internal Medicine, University of Kentucky College of Medicine, Lexington, KY 40536, USA;; 2 Department of Dermatology, Xijing Hospital, the Fourth Military Medical University, Shannxi, Xi’an 710032, P.R. China;; 3 Departments of Neurobiology and; 4 Pathophysiology, School of Basic Medicine, Anhui Medical University, Hefei, Anhui 230032, P.R. China;; 5 Graduate Center for Toxicology, University of Kentucky College of Medicine, Lexington, KY 40536, USA

**Keywords:** apoptosis, carcinogenesis, protection, protein degradation, skin damage

## Abstract

Ultraviolet B (UVB) exposure causes damage to skin and represents the primary etiological agent for skin cancer formation. UVB induces DNA damage and apoptosis in epidermal cells. In this study, we demonstrated that UVB activated autophagy in JB6 epidermal cells, which was evident by the formation of LC3 puncta, the induction of LC3 lipidation, the increase in beclin 1 expression, and the decrease in the levels of p62. Autophagy appeared to be a protective response to UVB-induced damage because inhibition of autophagy exacerbated UVB-induced cell death, and stimulation of autophagy offered protection. Furthermore, we demonstrated that glycogen synthase kinase 3β (GSK3β) was involved in UVB-induced autophagy. UVB inhibited GSK3β activation by simultaneously enhancing phosphorylation at Ser^9^ and suppressing Tyr^216^ phosphorylation. GSK3β negatively regulated autophagy; overexpression of wild-type or S9A (constitutive-active) GSK3β mutant inhibited UVB-mediated autophagy, while overexpression of a dominant-negative K85R mutant enhanced UVB-mediated autophagy. Inhibition of GSK3β also offered protection against UVB-mediated damage. UVB activated AMP-activated protein kinase (AMPK), an important regulator of autophagy through the inhibition of GSK3β. Taken together, our results suggest that UVB-stimulated autophagy is a protective response for epidermal cells and is mediated by the GSK3β/AMPK pathway.

## Introduction

UV irradiation is a major environmental stressor that results in altered cutaneous functions: for example, induction of inflammation, dyspigmentation, premature aging, skin cancer, and attenuation of barrier function (1). The terrestrial solar UV spectrum can be divided into UVA (320–400 nm), UVB (280–320 nm), and UVC (200–280 nm). Wavelengths in the UVB region are absorbed into the epidermis of the skin and cause skin disorders such as the formation of sunburn cells (apoptotic cells) and skin cancer (2,3).

UVB causes DNA damage and apoptosis of epidermal cells, and the signaling pathways associated with UVB-induced cell death have been extensively investigated (3). However, the cellular self-protection system in which epidermal cells respond to UVB is unclear. Autophagy is an intracellular degrading and recycling process by which organelles, cytoplasmic components, and invading pathogens are delivered to lysosomes for degradation (4,5). This process recycles cellular components and produces building blocks under stress conditions/nutrient starvation; it also eliminates damaged proteins/organelles (4–6). Therefore, autophagy has been proposed to be a cellular self-protection process in response to stress conditions (7,8). However, some studies suggest that autophagy may contribute to cellular damage (7).

In this study, we examined the effect of UVB on autophagy in a mouse epidermal cell line (JB6). JB6 cells have been extensively used to study the mechanisms of epidermal cell transformation (9–11). UVB causes the death of JB6 cells in the form of apoptosis (12). We demonstrated that UVB activated autophagy in JB6 cells, and autophagy appeared to be a protective response to UVB-induced damage. Furthermore, we showed that GSK3β negatively regulated autophagy and inhibition of GSK3β also offered protection against UVB-induced cell death.

## Materials and methods

### Materials

LiCl, bafilomycin A1, wortmannin, rapamycin, 3-methyladenine (3-MA) and anti-actin antibody were purchased from Sigma (St. Louis, MO, USA). Anti-beclin 1 antibody was purchased from Abcam (Cambridge, MA, USA). Anti-LC3 antibody was purchased from Medical and Biological Laboratories (Nagoya, Japan). The antibodies directed against GSK3β, phospho-GSK3β, AMPK, phospho-AMPK and p62 were obtained from Cell Signaling Technology (Beverly, MA, USA).

### Cell culture and UVB irradiation

JB6 mouse epidermal cells (CI 41) were cultured in EMEM supplemented with 10% fetal bovine serum (FBS), 2 mM L-glutamine, 25 μg/ml gentamicin, 100 U/ml penicillin and 100 μg/ml streptomycin at 37°C with 5% CO_2_. The establishment of JB6 cell lines stably expressing wild-type GSK3β (WT), constitutively active GSK3β (S9A), and dominant-negative GSK3β (K85R) have been previously described (10). The cells were irradiated with a UVB lamp (UVP, 0–400 mJ/cm^2^) as previously described (13) and then incubated at 37°C for the indicated time.

### Detection of LC3 puncta

GFP-LC3 plasmid was a generous gift from Dr Gutterman (University of Texas MD Anderson Cancer Center, Houston, TX). Transfections were performed using Lipofectamine™ 2000 (Invitrogen, Carlsbad, CA, USA) according to the manufacturer’s protocol. At 24 h after transfection, cells were exposed to UVB irradiation and were examined by a fluorescence microscope at indicated times. The GFP-LC3 puncta/cell were quantified as previously described (14,15). For each group, twenty cells in randomly selected visual fields were counted. The experiment was replicated three times.

### Determination of cell viability

Cells were exposed to UVB irradiation at indicated dosages and incubated for 24 h. Cell viability was determined by MTT assay as previously described (16).

### Immunoblotting

The procedure for immunoblotting has been previously described (10). Briefly, cells were washed twice with PBS and lysed with RIPA buffer [150 mM NaCl, 0.1% sodium dodecyl sulfate (SDS), 50 mM Tris (pH 8.0), 0.5% deoxycholic acid sodium, 1% Nonidet P-40 (NP-40), 0.1 mg/ml phenylmethylsulfonyl fluoride, 3% aprotinin, and 1 mM sodium orthovanadate] on ice for 10 min. Cell lysates were centrifuged at 12,000 rpm at 4°C for 10 min. The supernatant was then collected and the protein concentration was measured with a protein assay kit (Bio-Rad Laboratories, Hercules, CA, USA). An aliquot of the total protein (40 μg) was loaded into each lane of an SDS-polyacrylamide gel. The protein was electrophoretically transferred to nitrocellulose membranes and blocked with 5% BSA in 0.01 M TBS (pH 7.4) and 0.05% Tween-20 (TBST) at room temperature for 1 h. The blots were probed with primary antibodies for 2 h at room temperature or overnight at 4°C. After three quick washes with TBST, the membranes were incubated with horseradish peroxidase-conjugated goat anti-rabbit or goat anti-mouse IgG (Amersham, Arlington Heights, IL, USA) for 1 h and the bands were visualized with the enhanced chemiluminescence method (Amersham).

### Statistical analysis

All the data are expressed as the mean ± SD from at least three independent experiments. The statistical analysis was performed using the analysis of variance (ANOVA) followed by post hoc analyses. A value of p<0.05 was considered statistically significant.

## Results

### UVB irradiation activates autophagy

**Figure 1.**
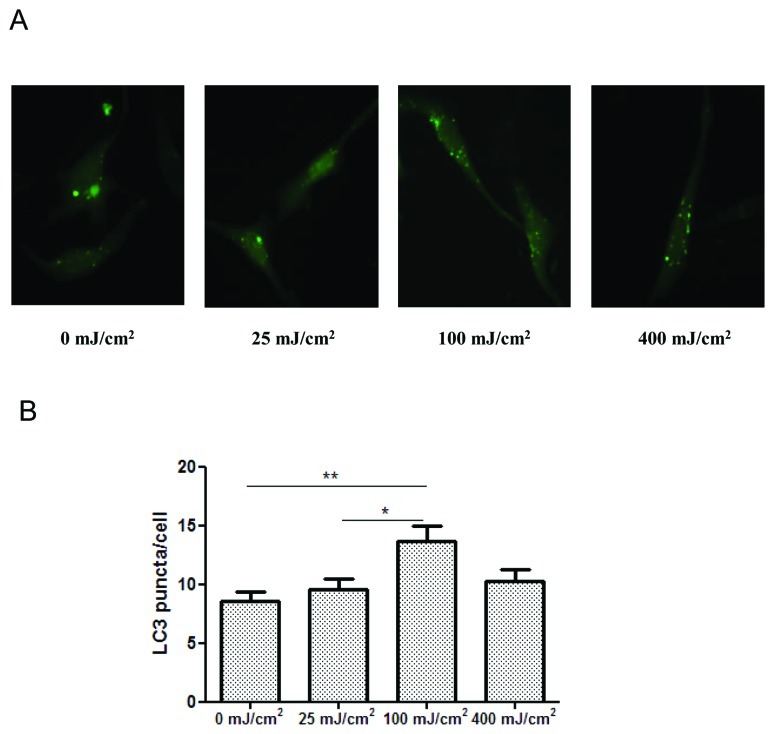

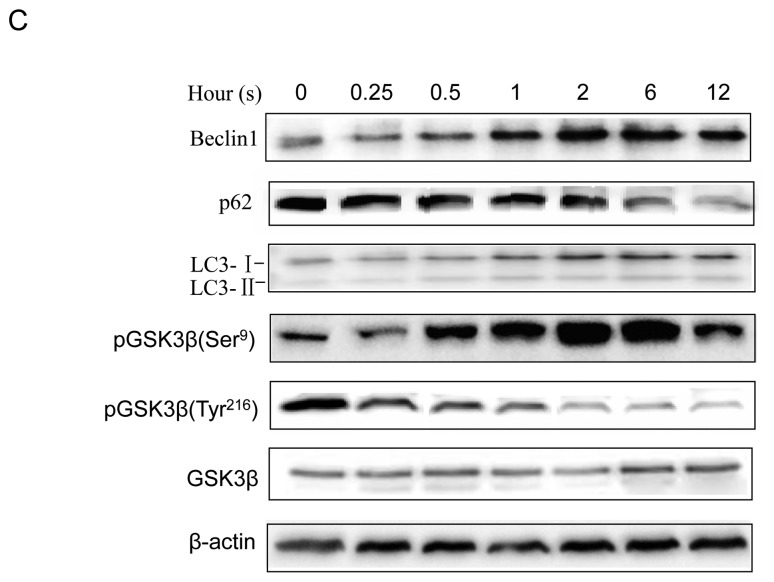
Effect of UVB on autophagy in JB6 cells. (A) JB6 cells were transfected with a GFP-LC3 plasmid and exposed to UVB irradiation (25 mJ/cm^2^, 100 mJ/cm^2^, or 400 mJ/cm^2^). The formation of GFP-LC3 punctate dots was visualized by a fluorescent microscope after incubation for 6 h. (B) The number of GFP-LC3 puncta/cell was quantified as described under the Materials and methods. The experiment was replicated three times. ^*^ and ^**^ denote statistically significant from the control group (^*^p<0.05; ^**^p<0.01). (C) JB6 cells were exposed to UVB (100 mJ/cm^2^) irradiation at specified times. The expression of p62, beclin 1, LC3 (LC3-I and LC3-II), GSK3β, and phosphorylated GSK3β (Ser^9^ and Tyr^216^) was detected with immunoblotting. The expression of actin served as a loading control. The experiment was replicated three times.

First, we examined the effect of UVB irradiation on autophagy in cultured JB6 mouse epidermal cells. The synthesis and conversion of LC3 is connected closely with the level of autophagy, making it a key marker in cells (17). The formation of LC3 puncta is an important indication of autophagy. We transfected JB6 cells with a GFP-LC3 plasmid, then examined the distribution and amount of green fluorescence LC3 puncta. We determined the effect of UVB irradiation at 0, 25, 100, 400 mJ/cm^2^ after 24 h of exposure. As shown in Fig. 1, a significant increase of LC3-GFP puncta formation was observed after UVB exposure at 100 mJ/cm^2^. UVB irradiation at 25 or 400 mJ/cm^2^ did not alter LC3 puncta. LC3 lipidation, which is indicated by the formation of LC3-II, is an index of autophagy. We demonstrated that UVB irradiation (100 mJ/cm^2^) increased the level of LC3-II (induction of LC3 lipidation) in JB6 cells in a time-dependent manner (Fig. 1C). Meanwhile, the expression of beclin 1 was upregulated, but the level of p62 was downregulated (Fig. 1C). Beclin 1, also known as Atg6, is a protein required for the formation of the initial autophagic structure, while p62 is regulated by autophagy-dependent degradation. Together, these results suggested that UVB irradiation activated autophagy.

**Figure 2.**
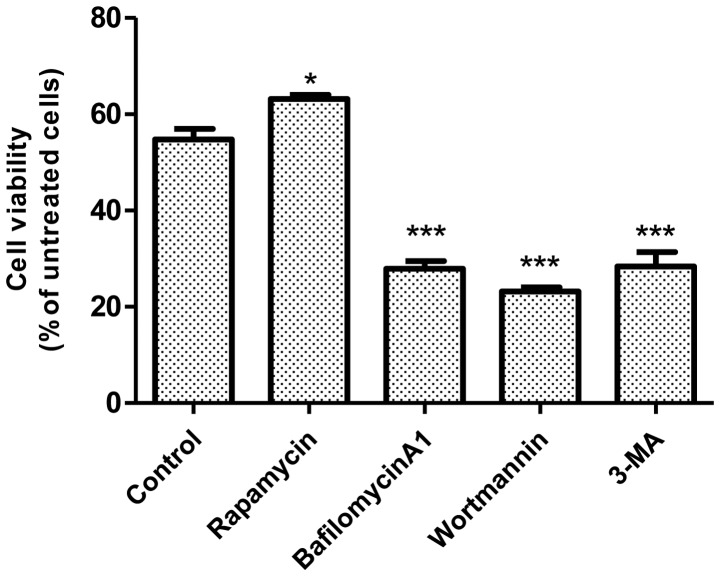
Effect of modulation of autophagy on UVB-induced cell death. JB6 cells were pretreated with rapamycin (10 nM), bafilomycin A1 (10 nM), wortmannin (10 μM), or 3-MA (5 mM) for 2 h and then irradiated by UVB (100 mJ/cm^2^). After 24-h incubation at 37°C, cell viability was determined by MTT assay as described under the Materials and methods. The experiment was replicated three times. ^*^ and ^***^ denote statistically significant from the control group (^*^p<0.05; ^***^p<0.001).

We next sought to determine the role of autophagy in UVB irradiation-induced cell death. As shown in Fig. 2, activation of autophagy by rapamycin offered protection, whereas inhibition of autophagy by bafilomycin A1, wortmannin, or 3-MA exacerbated UVB irradiation-induced cell death. These results suggested that autophagy was a protective response to UVB irradiation-induced damage.

### The involvement of GSK3β in UVB-activated autophagy

**Figure 3.**
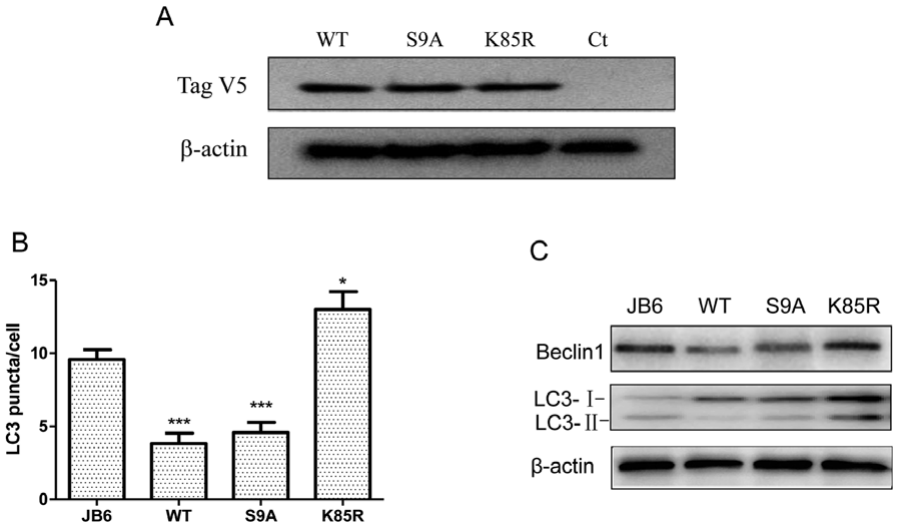
Effect of UVB on autophagy in JB6 cells stably expressing GSK3β mutants. (A) The expression of wild-type (WT), S9A and K85R GSK3_β_ in JB6 cells was confirmed by the expression of a V5 tag by immunoblotting. (B) JB6 cells stably expressing WT, S9A, and K85R GSK3β were transfected with a GFP-LC3 plasmid for 24 h. After that, cells were treated with UVB irradiation (100 mJ/cm^2^) and incubated for 6 h. The number of GFP-LC3 puncta/cell was quantified as described above. The experiment was replicated three times. ^*^ and ^***^ denote statistically significant from the control group (^*^p<0.05; ^***^p<0.001). (C) JB6 cells stably expressing wild-type, S9A, and K85R GSK3β were exposed to UVB irradiation (100 mJ/cm^2^), and cell lysates were collected after 6 h of incubation. The levels of LC3 and beclin 1 were analyzed by immunoblotting. The expression of actin served as a loading control. The experiment was replicated three times.

**Figure 4.**
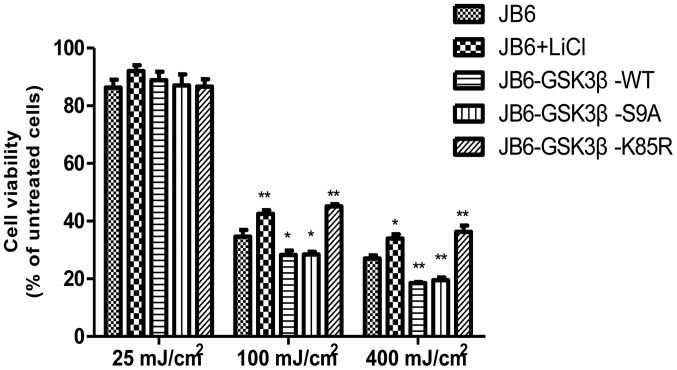
Effect of UVB on the viability of JB6 cells expressing GSK3β mutants. JB6 cells stably expressing WT, S9A and K85R GSK3β were exposed to UVB irradiation (25 mJ/cm^2^, 100 mJ/cm^2^, or 400 mJ/cm^2^). In some groups, JB6 cells were pretreated with LiCl (20 mM) for 1 h and then received UVB irradiation. After 24 h, cell viability was determined by MTT assay as described under the Materials and methods. The experiment was replicated three times. ^*^ and ^**^ denote statistically significant from the control group (^*^p<0.05 and ^**^p<0.01).

The activity of GSK3β is negatively regulated by the phosphorylation at Ser^9^, but positively at Tyr^216^(18). We showed that UVB irradiation increased the level of pGSK3β (Ser^9^) and decreased pGSK3β (Tyr^216^), indicating it inhibited GSK3β activity (Fig. 1C). To evaluate the role of GSK3β in UVB-induced autophagy, we established JB6 cells stably expressing various GSK3β mutants. These constructs included wild-type (WT), constitutive-active (S9A) and dominant-negative (K85R) GSK3β. S9A mutant is resistant to inhibitory regulation by restraining phosphorylation at Ser^9^; K85R mutant represents a deficit kinase and functions as a dominant-negative protein. We have previously shown they effectively stimulated or inhibited GSK3β activity, respectively (10). Overexpression of these exogenous GSK3β proteins was verified by the expression of a V5 tag by immunoblotting (Fig. 3A). We demonstrated that manipulation of GSK3β activity altered UVB-mediated autophagy. Upon UVB exposure (100 mJ/cm^2^), overexpression of WT and S9A GSK3β significantly decreased the number of LC3 puncta, whereas K85R GSK3β increased the amount of LC3 puncta (Fig. 3B). Consistent with this result, overexpression of WT and S9A GSK3β decreased the level of LC3-II and beclin 1; overexpression of K85R increased the expression of LC3-II and beclin 1. Together, these data suggested that inhibition of GSK3β resulted in an increase in autophagy. In parallel, overexpression of WT and S9A GSK3β exacerbated UVB-induced cell death, whereas overexpression of K85R GSK3β protected cells against UVB-induced cell death (Fig. 4). Protection mediated by the inhibition of GSK3β was further supported by a study using lithium treatment. Lithium, an inhibitor of GSK3β, reduced UVB-mediated cell death (Fig. 4).

### Interaction between AMPK and GSK3β in response to UVB

**Figure 5.**
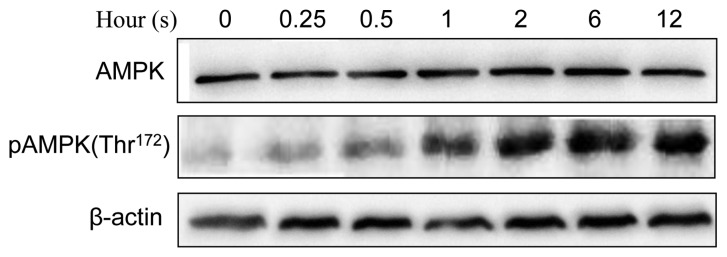
Effect of UVB on AMPK phosphorylation. JB6 cells were treated with UVB irradiation (100 mJ/cm^2^). The protein samples were collected at specified times. The expression of AMPK and phosphorylated AMPK (Thr^172^) was determined by immunblotting. The expression of actin served as a loading control. The experiment was replicated three times.

**Figure 6.**
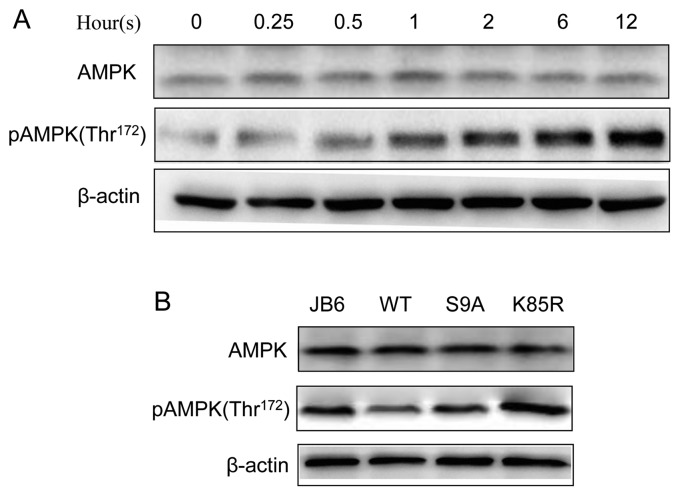
Role of GSK3β in UVB-mediated activation of AMPK. (A) JB6 cells were pretreated with LiCl (20 mM) for specified times. The expression of AMPK and pAMPK (Thr^172^) was examined with immunoblotting. The expression of actin served as a loading control. The experiment was replicated three times. (B) JB6 cells stably expressing wild-type, S9A and K85R GSK3β were exposed to UVB irradiation (100 mJ/cm^2^) and incubated for 6 h. The expression of AMPK and pAMPK (Thr^172^) was examined with immunoblotting. The expression of actin served as a loading control. The experiment was replicated three times.

AMPK is a critical regulator of autophagy and activation of AMPK results in enhanced autophagy (19). We confirmed that UVB induced the phosphorylation of AMPK (Thr^172^) in JB6 cells (Fig. 5). We further investigated the relationship between AMPK and GSK3β. Inhibition of GSK3β by lithium was sufficient to activate AMPK (Fig. 6A). Furthermore, overexpression of dominant-negative K85R GSK3β enhanced UVB-stimulated phosphorylation of AMPK (pAMPK), whereas overexpression of S9A or WT GSK3β inhibited UVB-mediated pAMPK (Fig. 6B). These results suggested that GSK3β negatively regulated AMPK phosphorylation.

## Discussion

In this study, we demonstrate for the first time that UVB can activate autophagy in epidermal cells, and autophagy appears to be a cytoprotective response to UVB-mediated damage.

UVB irradiation causes DNA damage and apoptosis of epidermal cells, contributing to human skin cancer in the process of tumor initiator and promoter (20,21). Mouse JB6 cells are a well-established *in vitro* model to study UVB-mediated damage and transformation of epidermal cells (22–24). Using this model, we demonstrate that UVB-induced reduction in the viability of JB6 cells is accompanied by the increase of autophagy which is evident by the formation of LC3 puncta, induction of LC3 lipidation, increase in beclin 1 expression, and decrease in the level of p62. Inhibition of autophagy by bafilomycin A1, wortmannin, or 3-MA exacerbates UVB-induced cell death. In contrast, activation of autophagy by rapamycin protects JB6 cells against UVB-mediated damage. This finding is consistent with a previous study showing that UV irradiation induced autophagy in A549 and H1299 cells (25,26). In that study, autophagy also seemed to be cytoprotective, and inhibition of autophagy exacerbated UV-triggered apoptotic cell death in these cells (26). Similarly, autophagy was shown to be cytoprotective against apoptosis induced by DNA-damaging agents (25).

It is interesting to note that UVB induces autophagy in a dose-dependent manner. At a low dosage, such as 25 mJ/cm^2^, UVB does not affect cell viability and autophagy. At 100 mJ/cm^2^, it causes cell death and activates autophagy. However, at a higher dosage, 400 mJ/cm^2^, it produces more cell death, but fails to activate autophagy (Fig. 1). It is likely that at a high dosage, UVB impairs autophagic machineries. This possibility remains to be investigated.

Another important finding for this study is that glycogen synthase kinase 3β (GSK3β) is involved in UVB-induced autophagy. GSK3β, a serine/threonine protein kinase, which was first described in glycogen metabolism and insulin signaling (27,28), is involved in multiple biological events such as embryonic development, stem cell survival, differentiation, neurodegeneration, tumorigenesis, and cell death (18,29,30). We have previously shown that inhibition of GSK3β promotes the transformation of epidermal cells (10). GSK3β activity is regulated by site-specific phosphorylation. The activity of GSK3β is upregulated by phosphorylation on the Tyr^216^ residue, and conversely, phosphorylation on Ser^9^ inhibits GSK3β activity. Phosphorylation of Ser^9^ is mediated by a number of signaling pathways, such as PI3K/AKT, PKC, MAPK/p90RS, or mTOR/p70S6 (18,31). The mechanism for the regulation of phosphorylation at Tyr^216^ is less clear. We demonstrate that UVB increases GSK3β phosphorylation at Ser^9^ but inhibits its phosphorylation at Tyr^216^, indicating that UVB inhibits GSK3β activity. UVB is shown to activate MAPK, PKC, and PI3K/AKT signaling pathways (32). It is therefore likely that UVB-induced phosphorylation of Ser^9^ is mediated by one or some of these pathways. Regardless of the mechanisms in which UVB inhibits GSK3β, it is likely that UVB activates autophagy through the inhibition of GSK3β because dominant-negative GSK3β enhances UVB-induced autophagy, whereas overexpression of GSK3β inhibits UVB-induced autophagy (Fig. 3). These results suggest that GSK3β negatively regulates autophagy and UVB may affect autophagy by modulating GSK3β activity.

AMP-activated protein kinase (AMPK), a crucial stress-sensing enzyme, is activated by a rise in the cellular AMP/ATP ratio. AMPK is an important mediator of autophagy (19). It has been demonstrated that activation of AMPK results in autophagy in human keratinocytes (33). Cadmium-induced activation of AMPK causes autophagy in JB6 cells (34). UV irradiation can regulate AMPK activity. For example, UVB is reported to activate AMPK in murine basal cell carcinoma and skin keratinocytes (35,36). UVC is shown to activate AMPK in pancreatic cancer cells (37). However, Zhang and Bowden (38) suggest that UVB inhibits AMPK in human keratinocytes. We demonstrate here that UVB activates AMPK in JB6 cells, and therefore UVB-mediated autophagy is regulated by AMPK (Fig. 5).

There is considerable interaction between GSK3β and AMPK (39–42). The interaction appears to be two-way; that is, AMPK can affect GSK3β activity and is also regulated by GSK3β. In our system, it appears that inhibition of GSK3β results in AMPK activation (Fig. 6). More importantly, inhibition of GSK3β by a dominant-negative GSK3β potentiates UVB-mediated AMPK activation, while overexpression of GSK3β inhibits UVB-induced AMPK activation. This is consistent with the effect of UVB/GSK3β on autophagy and supports a role of AMPK in UVB-mediated autophagy.

UVB causes apoptosis in JB6 cells through complex mechanisms; it may be mediated by oxidative stress, PKC, or a p53-dependent manner (12,43,44). The signaling pathways for UVB-mediated autophagy and apoptosis may or may not overlap. Our study indicates the GSK3β/AMPK pathway contributes to UVB-mediated autophagy. The interaction or cross-talk between the pathways governing autophagy and apoptosis in response to UVB remain to be studied.

## References

[1] Pustisek N, Situm M (2011). UV-radiation, apoptosis and skin. Coll Antropol.

[2] Matsumura Y, Ananthaswamy HN (2004). Toxic effects of ultra-violet radiation on the skin. Toxicol Appl Pharmacol.

[3] Lippens S, Hoste E, Vandenabeele P, Agostinis P, Declercq W (2009). Cell death in the skin. Apoptosis.

[4] Alers S, Löffler AS, Wesselborg S, Stork B (2012). The incredible ULKs. Cell Commun Signal.

[5] Palumbo S, Comincini S (2012). Autophagy and ionizing radiation in tumors: the ‘Survive or not Survive’ dilemma. J Cell Physiol.

[6] Mizushima N, Komatsu M (2011). Autophagy: renovation of cells and tissues. Cell.

[7] Chen N, Karantza-Wadsworth V (2009). Role and regulation of autophagy in cancer. Biochim Biophys Acta.

[8] Martínez-Borra J, López-Larrea C (2012). Autophagy and self-defense. Adv Exp Med Biol.

[9] Dong Z, Birrer MJ, Watts RG, Matrisian LM, Colburn NH (1994). Blocking of tumor promoter-induced AP-1 activity inhibits induced transformation in JB6 mouse epidermal cells. Proc Natl Acad Sci USA.

[10] Ma C, Wang J, Gao Y, Gao TW, Chen G, Bower KA, Odetallah M, Ding M, Ke Z, Luo J (2007). The role of glycogen synthase kinase 3beta in the transformation of epidermal cells. Cancer Res.

[11] Zhang D, Li J, Gao J, Huang C (2009). c-Jun/AP-1 pathway-mediated cyclin D1 expression participates in low dose arsenite-induced transformation in mouse epidermal JB6 C141 cells. Toxical Appl Pharmacol.

[12] Won YK, Ong CN, Shen HM (2005). Parthenolide sensitizes ultraviolet (UV)-B-induced apoptosis via protein kinase C-dependent pathways. Carcinogenesis.

[13] Song L, Gao M, Dong W, Hu M, Li J, Shi X, Hao Y, Li Y, Huang C (2011). p85α mediates p53 K370 acetylation by p300 and regulates its promoter-specific transactivity in the cellular UVB response. Oncogene.

[14] Alexander A, Cai SL, Kim J, Nanez A, Sahin M, Maclean KH, Inoki K, Guan KL, Shen J, Person MD, Kusewitt D, Mills GB, Kastan MB, Walker CL (2010). ATM signals to TSC2 in the cytoplasm to regulate mTORC1 in response to ROS. Proc Natl Acad Sci USA.

[15] Chen G, Ke Z, Xu M, Liao M, Wang X, Frank JA, Bower KA, Shi X, Luo J Autophagy is a protective response to ethanol neurotoxicity. Autophagy.

[16] Xu M, Bower KA, Wang S, Frank JA, Chen G, Ding M, Wang S, Shi X, Ke Z, Luo J (2010). Cyanidin-3-glucoside inhibits ethanol-induced invasion of breast cancer cells overexpressing ErbB2. Mol Cancer.

[17] Barth S, Glick D, Macleod KF (2010). Autophagy: assays and artifacts. J Pathol.

[18] Luo J (2009). Glycogen synthase kinase 3beta (GSK3beta) in tumorigenesis and cancer chemotherapy. Cancer Lett.

[19] Mihaylova MM, Shaw RJ (2011). The AMPK signaling pathway coordinates cell growth, autophagy and metabolism. Nat Cell Biol.

[20] Ehrhart JC, Gosselet FP, Culerrier RM, Sarasin A (2003). UVB-induced mutations in human key gatekeeper genes governing signalling pathways and consequences for skin tumourigenesis. Photochem Photobiol Sci.

[21] Xu Y, Shao Y, Zhou J, Voorhees JJ, Fisher GJ (2009). Ultraviolet irradiation-induces epidermal growth factor receptor (EGFR) nuclear translocation in human keratinocytes. J Cell Biochem.

[22] Huang C, Ma WY, Dong Z (1999). The extracellular-signal-regulated protein kinases (Erks) are required for UV-induced AP-1 activation in JB6 cells. Oncogene.

[23] Won YK, Ong CN, Shi X, Shen HM (2004). Chemopreventive activity of parthenolide against UVB-induced skin cancer and its mechanisms. Carcinogenesis.

[24] Roy S, Deep G, Agarwal C, Agarwal R (2012). Silibinin prevents ultraviolet B radiation-induced epidermal damages in JB6 cells and mouse skin in a p53-GADD45alpha-dependent manner. Carcinogenesis.

[25] Rodriguez-Rocha H, Garcia-Garcia A, Panayiotidis MI, Franco R (2011). DNA damage and autophagy. Mutat Res.

[26] Chen LH, Chu PM, Lee YJ, Tu PH, Chi CW, Lee HC, Chiou SH (2012). Targeting protective autophagy exacerbates UV-triggered apoptotic cell death. Int J Mol Sci.

[27] Ougolkov AV, Billadeau DD (2006). Targeting GSK-3: a promising approach for cancer therapy?. Future Oncol.

[28] Forde JE, Dale TC (2007). Glycogen synthase kinase 3: a key regulator of cellular fate. Cell Mol Life Sci.

[29] Kim JW, Lee JE, Kim MJ, Cho EG, Cho SG, Choi EJ (2003). Glycogen synthase kinase 3 beta is a natural activator of mitogen-activated protein kinase/extracellular signal-regulated kinase kinase kinase1(MEKK1). J Biol Chem.

[30] Luo J (2009). GSK3beta in ethanol neurotoxcity. Mol Neurobiol.

[31] Ding Q, He X, Xia W, Hsu JM, Chen CT, Li LY, Lee DF, Yang JY, Xie X, Liu JC, Hung MC (2007). Myeloid cell leukemia-1 inversely correlates with glycogen synthase kinase-3beta activity and associates with poor prognosis in human breast cancer. Cancer Res.

[32] Nomura M, Kaji A, Ma WY, Zhong S, Liu G, Bowden GT, Miyamoto KI, Dong Z (2001). Mitogen- and stress-activated protein kinase 1 mediates activation of Akt by ultraviolet B irradiation. J Biol Chem.

[33] Tong X, Smith KA, Pelling JC (2012). Apigenin, a chemopreventive bioflavonoid, induces AMP-activated protein kinase activation in human keratinocytes. Mol Carcinog.

[34] Son YO, Wang X, Hitron JA, Zhang Z, Cheng S, Budhraja A, Ding S, Lee JC, Shi X (2011). Cadmium induces autophagy through ROS-dependent activation of the LKB1-AMPK signaling in skin epidermal cells. Toxical Appl Pharmacol.

[35] Byekova YA, Herrmann JL, Xu J, Elmets CA, Athar M (2011). Liver kinase B1 (LKB1) in thepathogenesis of UVB-induced murine basal cell carcinoma. Arch Biochem Biophys.

[36] Cao C, Lu S, Kivlin R, Wallin B, Card E, Bagdasarian A, Tamakloe T, Wang WJ, Song X, Chu WM, Kouttab N, Xu A, Wan Y (2009). SIRT1 confers protection against UVB-and H2O2-induced cell death via modulation of p53 and JNK in cultured skin keratinocytes. J Cell Mol Med.

[37] Adachi S, Yasuda I, Kawaquchi J, Yamauchi T, Nakashima M, Itani M, Nakamura M, Yoshioka T, Moriwaki H, Kozawa O (2011). Ultraviolet enhances the sensitivity of pancreatic cancer cells to gemcitabine by activation of 5′AMP-activated protein kinase. Biochem Biophys Res Commun.

[38] Zhang J, Bowden GT (2008). UVB irradiation regulates Cox-2 mRNA stability through AMPK and HuR in human keratinocytes. Mol Carcinog.

[39] Shin SM, Cho IJ, Kim SG (2009). Resveratrol protects mitochondria against oxidative stress through AMP-activated protein kinase-mediated glycogen synthase kinase-3beta inhibition downstream of poly (ADP-ribose) polymerase-LKB1 pathway. Mol Pharmacol.

[40] de Candia P, Minopoli G, Verga V, Gargiulo A, Vanoni M, Alberghina L (2011). Nutritional limitation sensitizes mammalian cells to GSK-3β inhibitors and leads to growth impairment. Am J Pathol.

[41] Yuan HD, Piao GC (2011). An active part of *Artemisia sacrorum* Ledeb. Suppresses gluconeogenesis through AMPK mediated GSK3β and CREB phosphorylation in human HepG2 cells. Biosci Biotechnol Biochem.

[42] Yuan HD, Kim do Y, Quan HY, Kim SJ, Jung MS, Chung SH (2012). Ginsenoside Rg2 induces orphan nuclear receptor SHP gene expression and inactivates GSK3β via AMP-activated protein kinase to inhibit hepatic glucose production in HepG2 cells. Chem Biol Interact.

[43] Chen N, Ma W, Huang C, Dong Z (1999). Translocation of protein kinase Cepsilon and protein kinase Cdelta to membrane is required for ultraviolet B-induced activation of mitogen-activated protein kinases and apoptosis. J Biol Chem.

[44] Yang S, Misner BJ, Chiu RJ, Meyskens FL (2007). Redox effector factor-1, combined with reactive oxygen species, plays an important role in the transformation of JB6 cell. Carcinogenesis.

